# Lentinan improves intestinal inflammation and gut dysbiosis in antibiotics-induced mice

**DOI:** 10.1038/s41598-022-23469-2

**Published:** 2022-11-15

**Authors:** Xiuyu Ji, Le Su, Ping Zhang, Qiulin Yue, Chen Zhao, Xin Sun, Kunlun Li, Xinli Liu, Song Zhang, Lin Zhao

**Affiliations:** 1 State Key Laboratory of Biobased Material and Green Papermaking, School of Bioengineering, Qilu University of Technology, Shandong Academy of Sciences, Jinan, 250353 China; 2 Jinan Hangchen Biotechnology Co., Ltd, Jinan, People’s Republic of China; 3 Shandong Chenzhang Biotechnology Co., Ltd, Jinan, People’s Republic of China

**Keywords:** Diseases, Gastroenterology, Health care, Medical research

## Abstract

Gut microbiota dysbiosis is already a global problem after antibiotic overuse. This study was to investigate the therapeutic effect of lentinan and the mechanism of recovery of intestinal inflammation on broad-spectrum antibiotic-driven gut microbial dysbiosis in mice. Gut microbiota was elucidated by the Illumina MiSeq platform. Gas chromatography/mass spectrometry was used to investigate short-chain fatty acid content. Colon histology, expression of tight-junction associated proteins and pro-inflammatory cytokines levels were evaluated. The results showed that the gut microbiota of diversity and richness were reduced and various taxonomic levels of the gut microbiota were perturbed after antibiotics gavage. The abundance of Firmicutes and Bacteroidetes shifted to Proteobacteria and increased the relative abundance of harmful microbiota (*Parabacteroides* and *Klebsiella*) post-antibiotics, whereas lentinan administration reversed the dysbiosis and increased beneficial microbiota, including S24-7, *Lactobacillus*, *Oscillospira*, *Ruminococcus* and *Allobaculum*. The concentrations of propionic acid and butyric acid were significantly increased by treatment with lentinan. And lentinan improved colon tissue morphology and reduced pro-inflammatory cytokines via altering NF-κB signaling pathway in antibiotic-driven gut microbial dysbiosis mice. Taken together, the results proved that lentinan can be used as a prebiotic and the result provided a theoretical basis for improving the clinical treatment of broad-spectrum antibiotics side effects.

## Introduction

Antibiotics are invaluable weapons to fight infectious diseases and have made great contributions to public health as a kind of essential life-saving drug. However, the improper use of antibiotics has led to a series of clinical complications, such as antibiotic resistance^1^, antibiotic-associated diarrhea^2^, superinfection^3^, and gut microbiota metabolic disorder^4^. And many evidence showed that broad-spectrum antibiotics, particularly, impacted the overall abundance of bacterial composition and promoted a rapid decline in diversity in vivo models^5^, such as metronidazole, ampicillin, clindamycin, and vancomycin^6–8^.

The gut microbiota influenced essential host functions by modulating multiple endocrines, neural, and immune pathways of the host, including digestion, energy metabolism, and inflammation^9^. Its composition and complexity provided a certain level of resilience against external perturbation. However, the intestinal microbiota is modifiabled by dietary change^10^. It has been reported that many polysaccharides interact with the gut microbiota to regulate host health in different diseases. Panax ginseng polysaccharides positively affected antibiotic-associated diarrhea by modulating gut microbiota in mice^11^. Cyclocarya paliurus polysaccharides improved liver inflammation through altering the gut microbiota composition in mice^12^. Chrysanthemum polysaccharides ameliorated ulcerative colitis by modulating the balance of intestinal microecology^13^.

Next, the excessive intake of antibiotics often promotes the release of pro-inflammatory cytokines and intestinal mucosal damage^14^. The changes in pro-inflammatory cytokines and intestinal mucosal were closely related to the NF-κB signaling pathway^14^. Many studies have shown that polysaccharides affected body health by regulating the NF-κB signaling pathway. Poria Cocos polysaccharides exerted immunomodulatory effects via the NF-κB signaling^15^. Aloe polysaccharides inhibited HaCaT cell proliferation through over-activation of the NF-κB signaling pathway^16^. Fucoidan had an anti-inflammatory effect on RAW 264.7 macrophages via Blocking NF-κB signal Transduction^17^.

Lentinan, a specific class of β-glucans, is one of the most important bioactive compounds in Lentinus edodes and it is composed of a β-(1, 3)-glucose backbone with two (1, 6)-β-glucose branches of each five glucose units^18,19^. Lentinan is known to ameliorate intestinal inflammation and have a potential therapeutic effect on digestive diseases^10^. Lentinan had a potential anti-inflammatory effect on LPS-induced intestinal inflammatory response of juvenile taimen (Hucho taimen, Pallas)^20^. Emerging data have demonstrated that lentinan-based oral nanoparticle-loaded Budesonide (NT/BUD-NPs) significantly alleviated Ulcerative Colitis by targeting ability for treatment^21^. Moreover, lentinan significantly improved gut microbiota dysbiosis by decreasing phylum Proteobacteria and Epsilonbacteraeota^22^.

Based on the evidence and studies on the role of polysaccharides in the recovery of antibiotic-driven gut microbial dysbiosis, we aimed to investigate the therapeutic effects of lentinan on the broad-spectrum antibiotics-induced gut microbial dysbiosis in mice. Furthermore, we explored the changes in intestinal histology, inflammatory responses, expression of tight-junction proteins and short-chain fatty acids (SCFAs) metabolism after exposuring to antibiotics and lentinan treatments.

## Results

### Physiological effects of lentinan on antibiotic-induced mice

**Figure 1:**
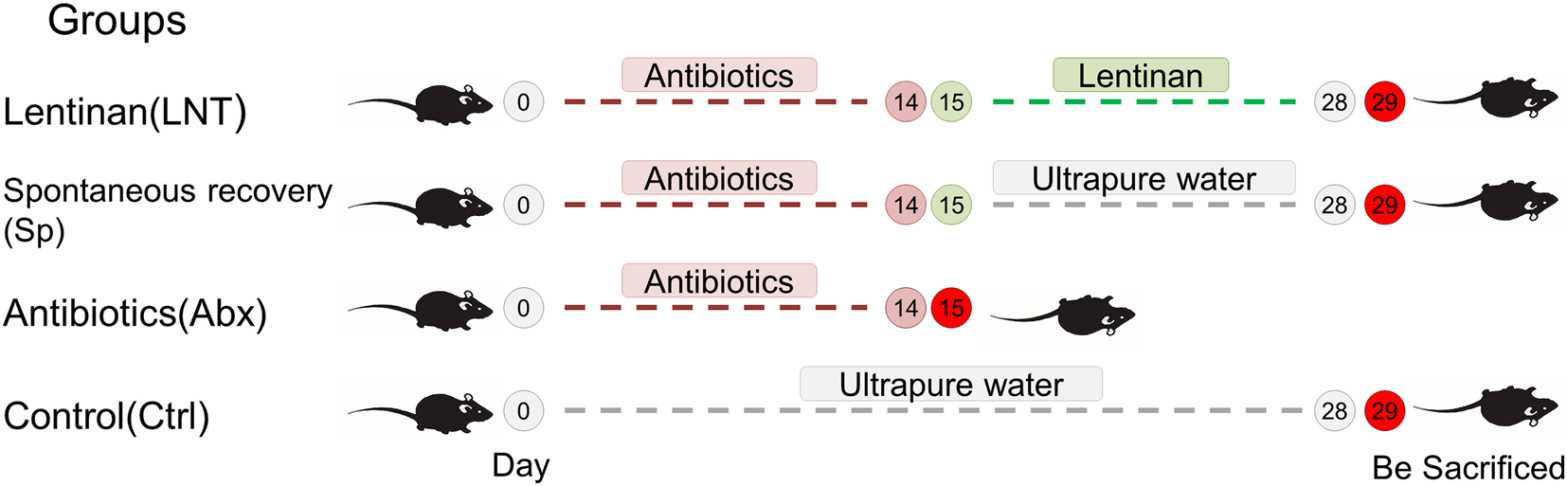
Experimental design of the study. Experimental plan outline where the control group (Ctrl, n = 5) received 200 μl/12 h ultrapure water from days 1 to 14 and 200 μl/24 h ultrapure water from days 15 to 28. The antibiotics groups (Abx, n = 5) received broad-spectrum antibiotics solution from days 1 to 14 and were sacrificed on day 15. The spontaneous recovery group (Sp, n = 5) received broad-spectrum antibiotics solution from days 1 to 14 followed by 200 μl/24 h ultrapure water from days 15 to 28 to observe spontaneous recovery. The lentinan group (LNT, n = 5) received broad-spectrum antibiotics solution from days 1 to 14 followed by lentinan treatment from days 15 to 28.

**Figure 2:**
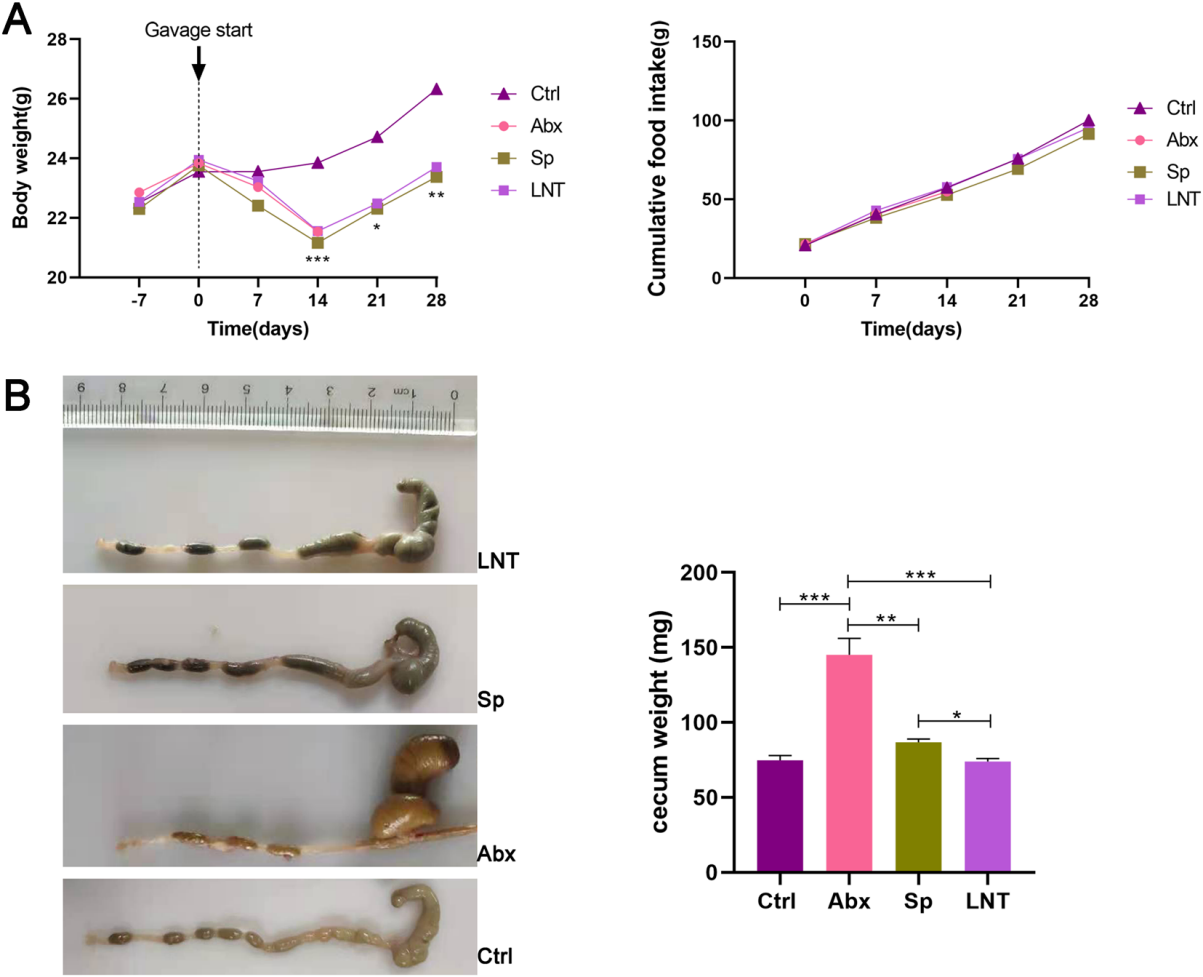
(**A**) Body weight measurement before and after the start of oral gavage in antibiotic-treated mice mode and Cumulative food consumption (g) after oral gavage. (**B**) The cecum size and weight of mice. Different groups by one-way ANOVA procedure followed by the Duncan test. *p < 0.05, **p < 0.01, ***p < 0.001. All error bars are SD.

In our study, dysbiosis was induced C57BL/6 J mice using the four broad-spectrum antibiotics ampicillin, vancomycin, metronidazole and neomycin sulfate clindamycin, and restoration was achieved by a lentinan fraction with the indicated concentration and time as illustrated (Fig. 1). The changes in body weight, cumulative food intake, cecum size, cecum weight, colon length and ileum length were displayed (Fig. 2 and Supplementary Fig. 1). After 14 days of antibiotics induction, the intestinal homeostasis was destroyed, the cecum was obviously swollen, and the body weight decreased significantly in mice.

We assessed the weight of each mouse during the treatment. The changes in body weight in mice of the control group were maintained at a steady level. And results revealed a significant reduction in the body weight at 2 weeks post-antibiotic treatment as compared to the Ctrl group (*** *p* < 0.001) (Fig. 2A). The weight of all mice showed an upward trend after antibiotic gavage was stopped. However, the lentinan group given lentinan treatment after 14 days showed no significant difference in body weight as compared to the spontaneous recovery group. Both Sp group and LNT group significant difference was observed as compared to Ctrl group after 21 days (* *p* < 0.05), and after 28 days (** *p* < 0.01).

We assessed the cumulative food intake of each group of mice during the treatment. The cumulative food consumption was indistinguishable among the four groups (Fig. 2A). And results revealed a significant augment to 2 times in the cecum size post-antibiotic treatment as compared to the Ctrl group (Fig. 2B). The cecum size of all mice showed a reverse trend after antibiotic gavage was stopped. LNT mice reduced the size of the cecum closer to Ctrl mice compared to Abx and Sp mice. Compared with the Ctrl group, colon length was shortened but not significantly different in Abx mice after antibiotics treatment (Supplementary Fig. 1A). There was no significant difference between the LNT group and the Sp group. And the ileum length was no significant difference among the four groups (Supplementary Fig. 1B). In general, there was no significant difference between LNT and Abx in terms of cumulative food intake, colon length and ileum length indexes.

### Histological characterization

**Figure 3:**
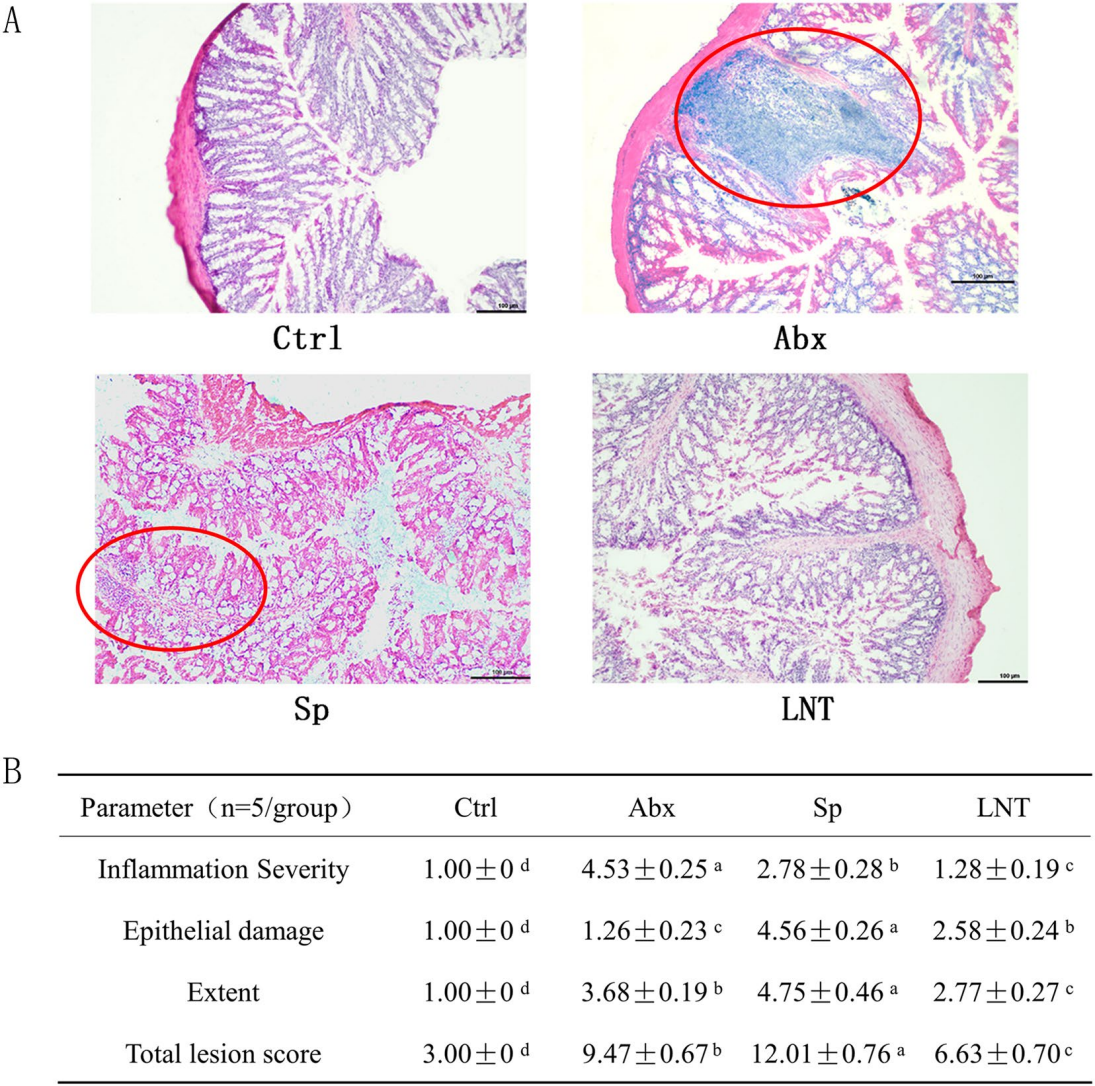
(**A**) Photomicrographs of hematoxylin and eosin (H&E)-stained colon tissue. Histopathological analysis of the colon tissue. The area of inflammation has been circled in red. Original magnification 100, Scale bar: 100 μm. (**B**) Effects of LNT on colon pathology of antibiotics-induced mice (n = 5/group). Values with different letters are significantly different (*p* < 0.05).

The histological evaluation of colonic tissue of mice in every group was done by hematoxylin and eosin (H&E) staining (Fig. 3). It revealed that the mice in the Ctrl group had normal colonic histology including no inflammatory response, no damage, and well-shaped villi. The mice in Abx group altered histology and were infiltrated by inflammatory cells. Those changes caused by antibiotics indicated that antibiotic exposure induced colonic inflammation. Sp mice reduced the number of inflammatory cells, but epithelial barrier and villi damaged. LNT mice reduced the number of inflammatory cells, and the villi of the colon were smoother and closer to Ctrl mice compared to Sp mice. The reduced the histological colon damage were reduced in the LNT group suggesting that lentinan significantly alleviated the pathological features of the colon and improved the severity extent of colon histological inflammation in the macroscopic. These results suggested that antibiotics caused colitis and lentinan significantly reduced the pathological features of the colon.

### Regulation of lentinan on the transcriptional expression of intestinal integrity genes

Broad-spectrum antibiotics alter the gut barrier function^5^. Intestinal permeability was correlated with the expression levels of pro-inflammatory cytokines and tight junction proteins. RT-qPCR was used to measure the transcriptional expression of ZO-1 and Occludin. Intestinal inflammation often is accompanied by changes in the intestinal tight junction. So the relative expression level of ZO-1 and Occludin in the colonic was considered in this study.

**Figure 4:**
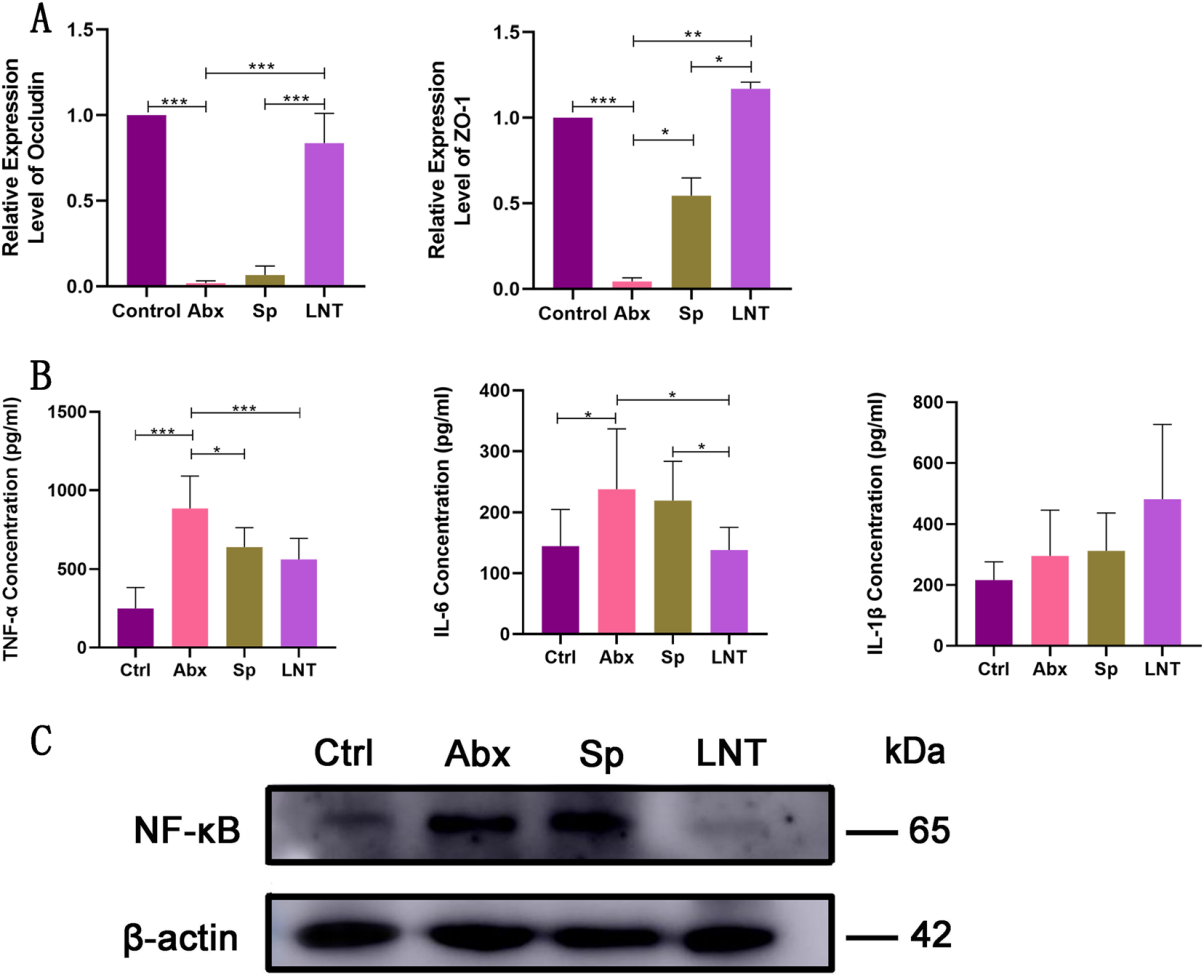
(**A**) The relative expression of intestinal tight junction (ZO-1 and Occludin) in the colon. (**B**) The levels of inflammatory cytokines (TNF-α, IL-6 and IL-1β) in the colon. (**C**) Expression of NF-κB in different groups using β-actin as an internal control. Western blots are representative of three independent experiments. Different groups by one-way ANOVA procedure followed by Duncan test. *p < 0.05, **p < 0.01, ***p < 0.001. All error bars are SD.

RT-qPCR results showed that the expression of intestinal tight junction proteins ZO-1 and Occludin were significantly lower after antibiotics gavage than Ctrl mice (Fig. 4A). Lentinan significantly increased the expression of intestinal tight junction proteins ZO-1 and Occludin after antibiotic-treated mice as compared to Sp mice. The results suggested that lentinan reversed the changes caused by broad-spectrum antibiotics in mice.

### Regulation of lentinan on the expression of inflammatory cytokines

Based on previous research, broad-spectrum antibiotics induced mice displayed colon histopathological features resembling inflammatory bowel disease^23^. Thereinto, pro-inflammatory cytokines mainly included TNF-α, IL-6 and IL-1β. ELISA was used to measure the levels of colonic TNF-α, IL-6 and IL-1β.

ELISA results showed that pro-inflammatory cytokines TNF-α, IL-6 and IL-1β were higher after antibiotics gavage (Fig. 4B). These results were also supported by H&E staining. Lentinan significantly reduced the levels of inflammatory cytokines TNF-α and IL-6 in antibiotics-treated mice as compared to Abx mice. Nonetheless, there was no significant change in IL-1β content between the LNT group and the Abx group.

### Regulation of lentinan on the expression of the signaling pathway

To analyze how antibiotics cause intestinal damage in mice, we determined whether the NF-κB signaling pathway was activated. NF-κB signaling pathway significantly up-regulated expression in the Abx mice compared with those in the Ctrl mice in western blot (Fig. 4C and Supplementary Fig. 5). The expression of NF-κB was significantly down-regulated in LNT mice compared with those in Sp mice. The expression of intestinal tight junction-associated proteins results also suggested significant therapeutic effects of lentinan. Spontaneous recovery was not as effective as lentinan in the results.

In a word, broad-spectrum antibiotics reduced the expression of tight junction proteins, increased the level of pro-inflammatory cytokines and activated the NF-κB signaling pathway. Lentinan reversed the changes caused by broad-spectrum antibiotics in mice.

### Lentinan modulated the composition and function of the gut microbiota in mice

**Figure 5:**
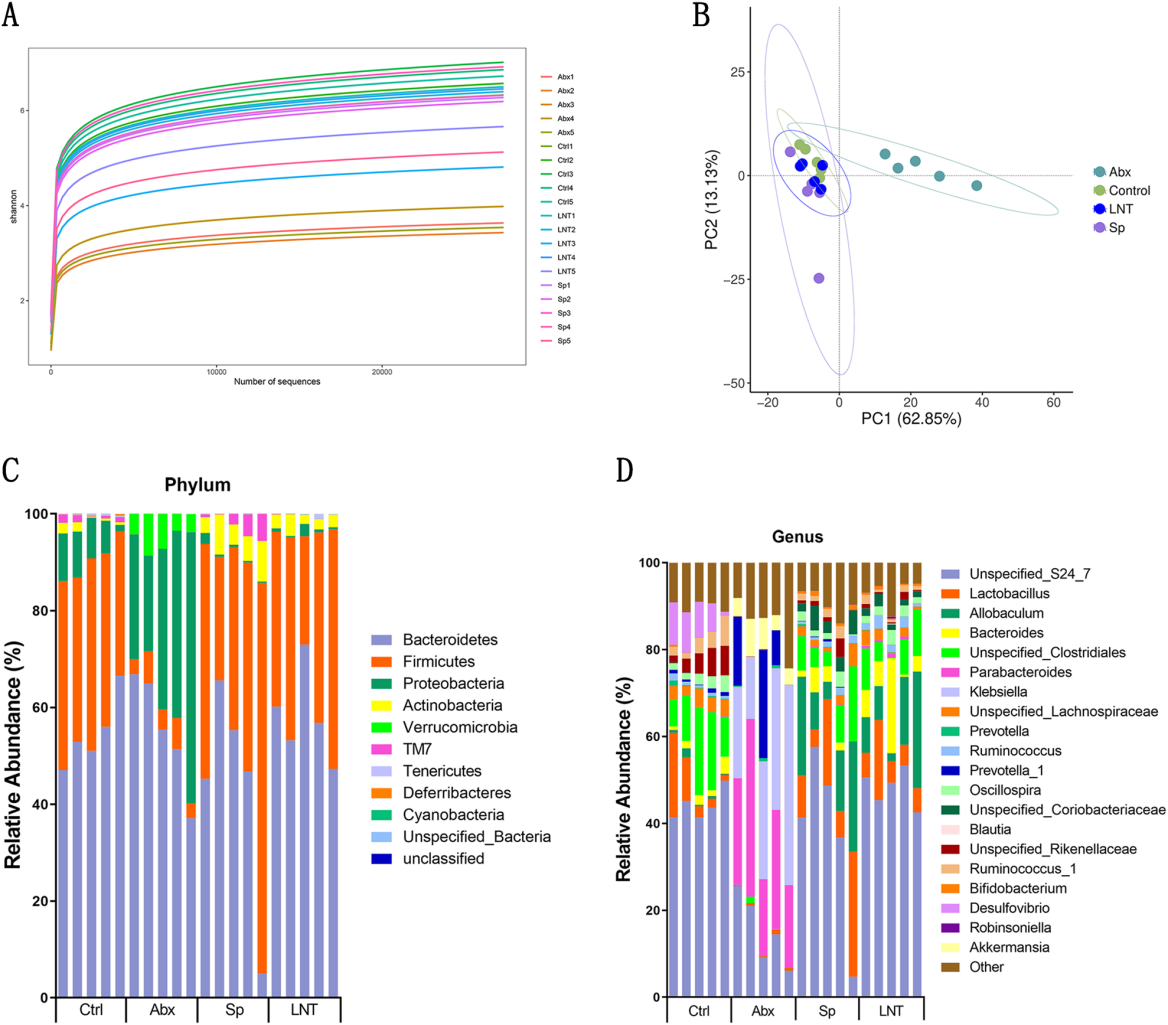
Effect of the gut microbiota. (**A**) Comparison of alpha diversity for mice. (**B**) Comparison of Beta diversity for mice. (**C**) Relative abundance of the Phyla and Genera levels.

Antibiotic treatment has altered the gut microbial relative abundance and diversity of mice as indicated by the Abx group compared to the Ctrl group. 16S rRNA gene sequencing results showed that LNT modulated the gut microbiota in antibiotic-induced mice (Fig. 5).

For comparing the total diversity and species richness in different treatment groups, we measured the alpha diversity of every sample (Fig. 5A and Supplementary Fig. 2A). The Shannon rarefaction curve showed that the sequencing depth was sufficiently credible and suitable for further analysis. Alpha diversity indexes are an analysis of species diversity, including the richness and evenness of species composition. Among alpha diversity, Shannon index means the evenness of samples, and Observed otus index means the richness of samples. From the perspective of Shannon index and Observed otus index, Abx mice resulted in a reduction in species evenness and richness compared to Ctrl mice. Alpha diversity of the gut microbiota showed no significant differences between the LNT group and the Sp group. The study data showed that species evenness and richness were reduced in antibiotics induced by the gut microbiota in mice. However, the LNT mice showed no significant change in the evenness and richness of the gut microbiota compared to Sp mice.

The main purpose of beta diversity analysis is to evaluate the differences among multiple groups. We mainly analyzed data structure differences through the principal component analysis (PCA) methods (Fig. 5B). The distance of samples demonstrated the indicative of similarities and independence among the four groups. The Unweighted UniFrac Principal coordinates analysis (Unweighted UniFrac PCoA) and Weighted UniFrac Principal coordinates analysis (Weighted UniFrac PCoA) methods are also represented by heat maps (Supplementary Fig. 3). Our results demonstrated that broad-spectrum antibiotics altered the beta diversity of the gut microbiota, and lentinan had an ameliorative effect on antibiotic-induced dysbiosis by recovering the similarities of microbiota closer to Ctrl mice.

At the phylum level, Firmicutes, Bacteroidetes, Proteobacteria, and Verrucomicrobia were the dominant phylum (Fig. 5C and Supplementary Fig. 4). The microbiome of Abx mice had a drastic decrease in the proportion of Firmicutes (Abx 4.72% vs. Ctrl 35.71%). The microbiome of Abx mice had a compositional shift to Proteobacteria (Abx 34.68% vs. Ctrl 7.14%) and Verrucomicrobia (Abx 5.48% vs. Ctrl 0.00%). An abnormal expansion of Proteobacteria made a compromised ability to maintain a balanced gut microbial community and an increase of Proteobacteria was considered a potential signature of dysbiosis and risk of disease^24,25^. In addition, the proliferation of Proteobacteria potential pathogenic species was observed in children with kwashiorkor^26^. After 2 weeks of antibiotic cessation, surprisingly the ratio of Firmicutes/Bacteroidetes in the Sp group and the LNT group almost returned to the initial level. Oral gavage of lentinan not only efficiently restored the proportion of the perturbed phylum but also decreased Proteobacteria (Abx 34.68% vs. Ctrl 7.14%).

At the genus level, dominant bacteria genera included *Unspecified S24-7*(Ctrl 44.27%, Abx 15.29%, Sp 37.83% and LNT 48.21%), *Lactobacillus* (Ctrl 7.13%, Abx 0.57%, Sp 6.57% and LNT 7.91%), *Ruminococcus* (Ctrl 0.86%, Abx 0.00%, Sp 1.14% and LNT 1.93%), *Allobaculum* (Ctrl 0.75%, Abx 0.00%, Sp 8.82% and LNT 12.04%), and *Oscillospira* (Ctrl 2.40%, Abx 0.00%, Sp 1.75% and LNT 2.19%) in Ctrl, Sp and LNT (Fig. 5D), while the Abx group showed obvious lower intensity or even vanish from sight. *Unspecified S24-7*, *Ruminococcus* and *Allobaculum* are SCFA-producing bacteria. The contents of SCFAs are related to host health. The relative abundances of Oscillospira were positively correlated with health^27^. *Lactobacillus* was a common intestinal probiotic to maintain ecological balance. The contents of these genera in the LNT group were higher than those in the Sp group. Posteriorly, the microbiome of Abx mice had a drastic increase in the proportion of compositional about *Parabacteroides* (Abx 25.9% vs. Ctrl 0.08%), *Klebsiella* (Abx 28.22% vs. Ctrl 0.00%) and *Akkermansia* (Abx 5.48% vs. Ctrl 0.00%). *Parabacteroides* and *Klebsiella* are harmful bacteria. *Akkermansia* was found to bloom after exposure to vancomycin in both animals and humans^28^. However, the contents of these genera in the LNT group were significantly reduced. Therefore, we believed that lentinan played an important role in the clinical significance via the regulation of gut microbial dysbiosis.

### Effects of lentinan on the production of SCFAs

**Figure 6:**
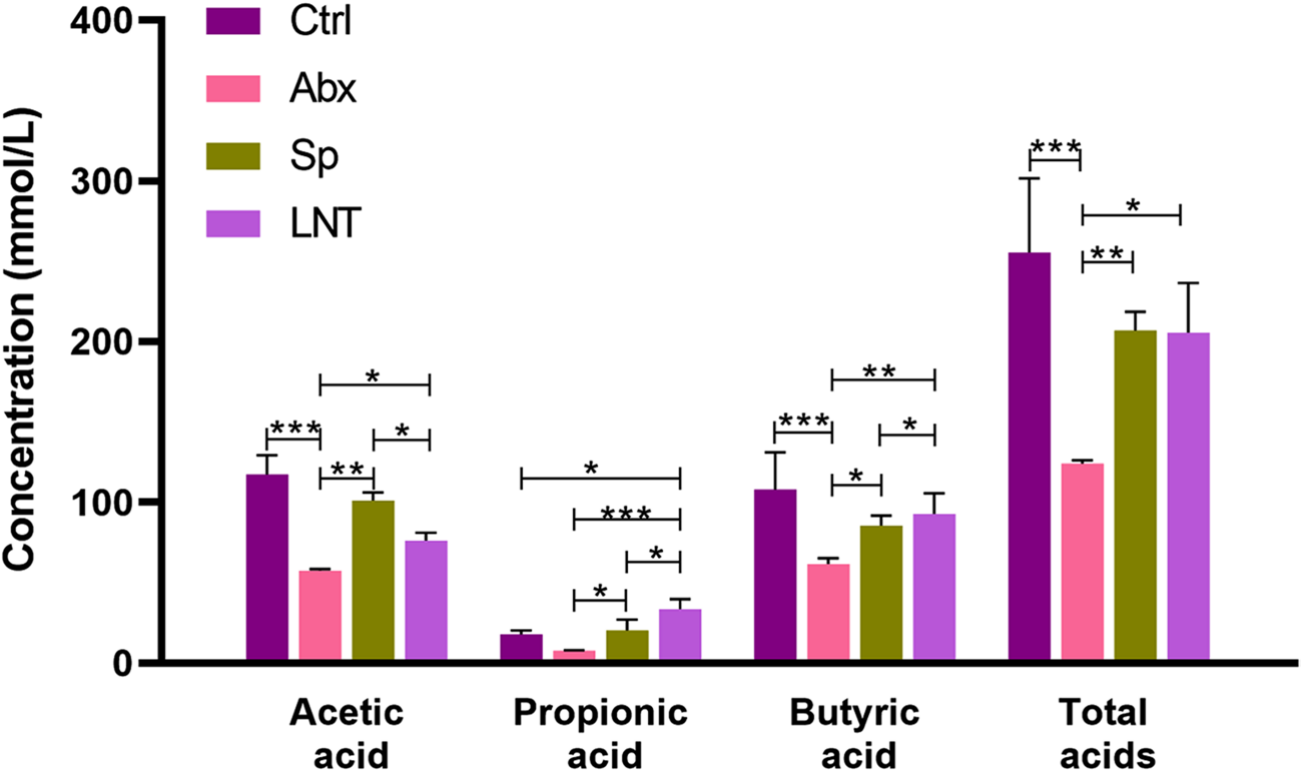
Effects of LNT on the production of SCFAs. Different groups by one-way ANOVA procedure followed by the Duncan test. *p < 0.05, **p < 0.01, ***p < 0.001. All error bars are SD.

SCFAs are metabolites of the gut microbiota in colon that were analyzed. Compared with the Ctrl mice, the concentrations of colon contents acetic acid, butyric acid, and total acid in the Abx group were significantly decreased (***p < 0.001) (Fig. 6). Acetic acid, propionic acid, butyric acid, and total acid concentrations in colon contents of Abx mice were reduced by half compared with Ctrl mice. Lentinan significantly improved the content of propionic acid and butyric acid in the colon compared to the Sp group (*p < 0.05). The content of propionic acid in colon was much lower than those of other acids in each group.

## Discussion

In this study, C57BL/6 J mice were induced by four broad-spectrum antibiotics, including metronidazole, ampicillin, clindamycin and vancomycin, to establish an antibiotic-driven gut microbial dysbiosis model, and we examined the regulatory effect of lentinan on gut microbiota community abundance and structure. Hippocrates, the father of modern medicine, believed that all disease begins in the gut^29^. A tight, balanced and orderly intestinal microecology in the gastrointestinal tract is the most stable foundation of host health. 16S rRNA data results showed that lentinan reversed the composition and function of the gut microbiota in antibiotic-driven gut microbial dysbiosis mice. It’s worth noting that the distribution of gut microbiota in the LNT group was closer to the Ctrl group compared to the Sp group. At the phylum level, the oral gavage of lentinan efficiently reversed the ratio of Firmicutes/Bacteroidetes, and decreased the abundance of Proteobacteria and Actinobacteria. At the genus level, the Abx group increased the abundance of *Parabacteroides*, *Klebsiella* and *Akkermansia* compared to the Ctrl group. *Parabacteroides* were resistant to a variety of antibiotics, including tetracycline and erythromycin^30^. *Akkermansia* was found to bloom after exposure to vancomycin in both animals and humans^28^. *Klebsiella* frequently caused nosocomial infections^31^. Elevated levels of *Klebsiella* in the intestinal microbiota were associated with a variety of diseases, such as depression^32^, inflammatory bowel disease (IBD)^33^, and so on. But lentinan decreased the abundance of these bacteria. The results indicated that lentinan effectively reverses the tumult caused by antibiotics and may reduce inflammation. The family *S24-7* plays a dominant role in the mouse gut microbiota^34^. *S24-7* was a producing SCFAs family^35^ and had a function of complex carbohydrate degradation by produce enzymes^36^. Both SCFAs and *S24-7* showed an upward trend in LNT group, so we regarded that *S24-7* may promote the production of SCFAs. The research confirmed that lentinan effectively restored the variation about gut microbiota caused by antibiotics. Based on the above changes in gut microbiota, we proved that lentinan restored intestinal health through affecting SCFAs metabolism by altering the composition of the gut microbiota.

According to a study, the change of SCFAs contents represented the homeostasis of intestinal microbiota^37^. SCFAs are important metabolites produced by intestinal microorganisms in the digestion of polysaccharides^38,39^. In our study, acetic acid, propionic acid, butyrate, and total acid concentrations in colon contents of Abx mice were reduced by half compared with Ctrl mice. The results confirmed earlier studies that antibiotics affected gut signaling by altering the prime metabolites of SCFAs^40^. Lentinan significantly improved the content of propionic acid and butyric acid in the colon compared to the Sp group. Propionic acid and butyric acid were absorbed to provide energy by intestinal epithelial cells and had various beneficial effects on host health^41^. Among them, the amount of butyric acid is inversely proportional to the development of colitis^42^. SCFAs regulated the secretion of inflammatory factors^37^. Therefore, lentinan restrained the expression of inflammatory factors by increasing the contents of SCFAs through altering the structure of the gut microbiota.

Many studies showed that antibiotics-associated dysbiosis with increased susceptibility to a myriad of chronic and infectious diseases^43,44^. The intestinal epithelial barrier is a site of action for recovering from antibiotic-driven gut microbial dysbiosis. Therefore, the influence of gut microbial dysregulation on the histological and molecular levels of the intestinal tract and its influencing mechanism deserves further study. From the results of the intestinal tissue section, the villi of the colon in mice of the LNT group were smoother and closer to rats of the Ctrl group than to those of the Sp group. The decrease of intestinal mucosal proteins like ZO-1 and Occludin expression was the key to intestinal barrier dysfunction^45^. Occludin was associated with epithelial stability^46^. ZO-1 was associated with epithelial integrity^47^. ZO-1 and Occludin in the LNT group were significantly increased compared with the Sp group. The expression of intestinal tight junction-associated proteins in RT-qPCR results also suggested significant therapeutic effects of lentinan. Destruction of the intestinal epithelial barrier may lead to inflammation, further causing bacterial translocations and passage of other pathogens^48^. The contents of TNF-α, IL-6 and IL-1β played important roles in the inflammatory situation^49^. A direct relationship has been reported between the contents of TNF-α, IL-6 and IL-1β and inflammatory situation^50,51^. LNT group increased the abundance of *Oscillospira* compared to the Sp group. The relative abundances of *Oscillospira* were positively correlated with health^27^ and negative correlation to the contents of inflammatory factors^52^. And lentinan significantly reduced the levels of pro-inflammatory cytokines TNF-α and IL-6 in antibiotics-treated mice. These results together indicated that LNT possessed the anti-inflammatory ability and the potential to reverse intestinal barrier function.

Research showed that the up-regulated expression of NF-κB was connected with antibiotics used^53^, which was consistent with this study. The NF-kB signaling pathway was an important pathway leading to inflammatory bowel disease. NF-κB mediated the release of pro-inflammatory factors^54^. And activation of the NF-κB signaling pathway promoted the release of pro-inflammatory factors^55,56^. A higher concentration of the TNF family led  to down-regulation of the expression of ZO-1 protein down-regulation^57^. The result was consistent with the contents of pro-inflammatory cytokines increased after antibiotics (Fig. 4B) and the expression of intestinal mucosal protein down-regulation (Fig. 4A). Meanwhile, the TNF family was an inducer activating the NF-κB signaling pathway^58,59^. Antibiotics increased the level of pro-inflammatory cytokines and reduced the expression of tight junction proteins by activating the NF-κB signaling pathway. In the meantime, antibiotics down-regulated the expression of tight junction proteins and activated the expression of NF-κB signaling pathway by increasing the level of pro-inflammatory cytokines. Nevertheless, lentinan can effectively reduce the harmful effects of antibiotics through inhibiting the expression of the NF-κB signaling pathway.

## Conclusion

Antibiotics perturbed the gut microbiota, broke the intestinal barrier and made pathogen overgrowth in the intestinal, which affected host homeostasis, SCFAs and intestinal permeability. Treatment with lentinan down-regulation the expression of NF-κB signaling pathway, increased the expression of tight-junction proteins, decreased pro-inflammatory cytokine levels and increased the contents of SCFAs through modulating gut microbiota. Lentinan supplementation restored the gut microbiota community structure, enhanced microbial diversity and reduced intestinal inflammation caused by antibiotics through the down-regulation NF-κB signaling pathway. Taken together, these results revealed that lentinan can be used as a prebiotic and has shown a potential intestinal protective effect by reverting the antibiotic-associated dysbiosis and related adverse effects.

## Materials and methods

### Materials

Lentinan (molecular weight, 36,000–50,000) was purchased from Solarbio Science & Technology Co., Ltd. (Beijing, China). The antibiotics ampicillin, neomycin sulfate, metronidazole and vancomycin were purchased from Shanghai Macklin Biochemical Co., Ltd. (Shanghai, China). The broad-spectrum antibiotics solution was comprised of four antibiotics (ampicillin (100 mg/kg, Macklin Biochemical), vancomycin (50 mg/kg, Macklin Biochemical), metronidazole (100 mg/kg, Macklin Biochemical), neomycin sulfate (100 mg/kg, Macklin Biochemical)) and made fresh every 24 h.

### Animal ethics statement

Male C57BL/6 J mice (7-week-old, 22.5 ± 0.7 g) were purchased from Charles River Laboratory Animal Technology (Beijing, China). All animals had free access to food and drinking water and were housed in a controlled room (temperature, 25 ± 2 °C; relative humidity, 45–60%; lighting cycle, 12 h/d; 06:00–18:00 for light) during the first 7 days acclimation period.

### Animals’ experimental design

After the acclimation period, 20 mice were distributed into four groups (n = 5 per group): the control group (Ctrl), the antibiotics group (Abx), the spontaneous recovery group (Sp) and the lentinan group (LNT). Ctrl mice received 200 μl ultrapure water every 12 h for 14 consecutive days and then received 200 μl ultrapure water every 24 h for 14 consecutive days (Fig. 1). Abx, Sp and LNT mice were given oral gavage of broad-spectrum antibiotics solution every 12 h for 14 consecutive days. Abx mice were euthanized by cervical dislocation 24 h after the last oral gavage of broad-spectrum antibiotics solution. Colon tissue, colon contents and cecum contents of all animals were collected and stored at − 80 °C for the following experiments. Thereafter, LNT mice were orally gavaged with lentinan (L8270, (C_42_H_70_O_35_)_n_) solution (200 mg/kg×day) for 14 consecutive days every morning. Sp mice received 200 μl ultrapure water every 24 h for 14 consecutive days to assess the spontaneous recovery of the indigenous gut microbiome in this setting. On day 29, other mice were sacrificed by cervical dislocation. Colon tissue, colon contents and cecum contents of all animals were collected and stored at − 80 °C for the following experiments.

### Histological observation of colon tissue

The distal colon tissues were dissected and immersed in an optimal cutting temperature compound (OCT) embedding medium (Tissue-Tek). Serial 10-μm-thick cryosections were mounted on poly-d-lysine-coated slides. Cryosections were stained with hematoxylin and eosin (H&E). Cryosections of colon tissue were observed under a Nikon Microscope (Tokyo, Japan). The intestinal inflammation was graded blindly by two observers^60^. The scoring system mainly comprised two parameters, tissue damage and infiltration of lamina propria by inflammatory cells, and scored from 1 (no changes) to 5 (widespread cellular infiltration and extensive tissue damage).

### RNA extraction and quantification of gene expression

Total RNAs of each full colonic tissue were prepared using the classic TRIzol method and quantified by using NanoPhotometer NP80 Touch (IMPLEN GMBH, Germany). cDNA was synthesized by reverse transcription of RNA by ABScript II RT Mix for qPCR with gDNA Remover (ABclonal RK20403, China). The forward and reverse primers of qPCR (Sangon Biotech, Shanghai, China) were designed and synthesized (Table 1). Quantitative real-time polymerase chain reaction (RT-qPCR) was performed with 2X Universal SYBR Green Fast qPCR Mix (ABclonal RK21203, China) according to the protocol of the manufacturer. The relative gene expression level of the target genes was calculated by 2^−∆∆Ct^, and glyceraldehyde-3-phosphate dehydrogenase (GAPDH) was used as the housekeeping gene.

**Table 1:**
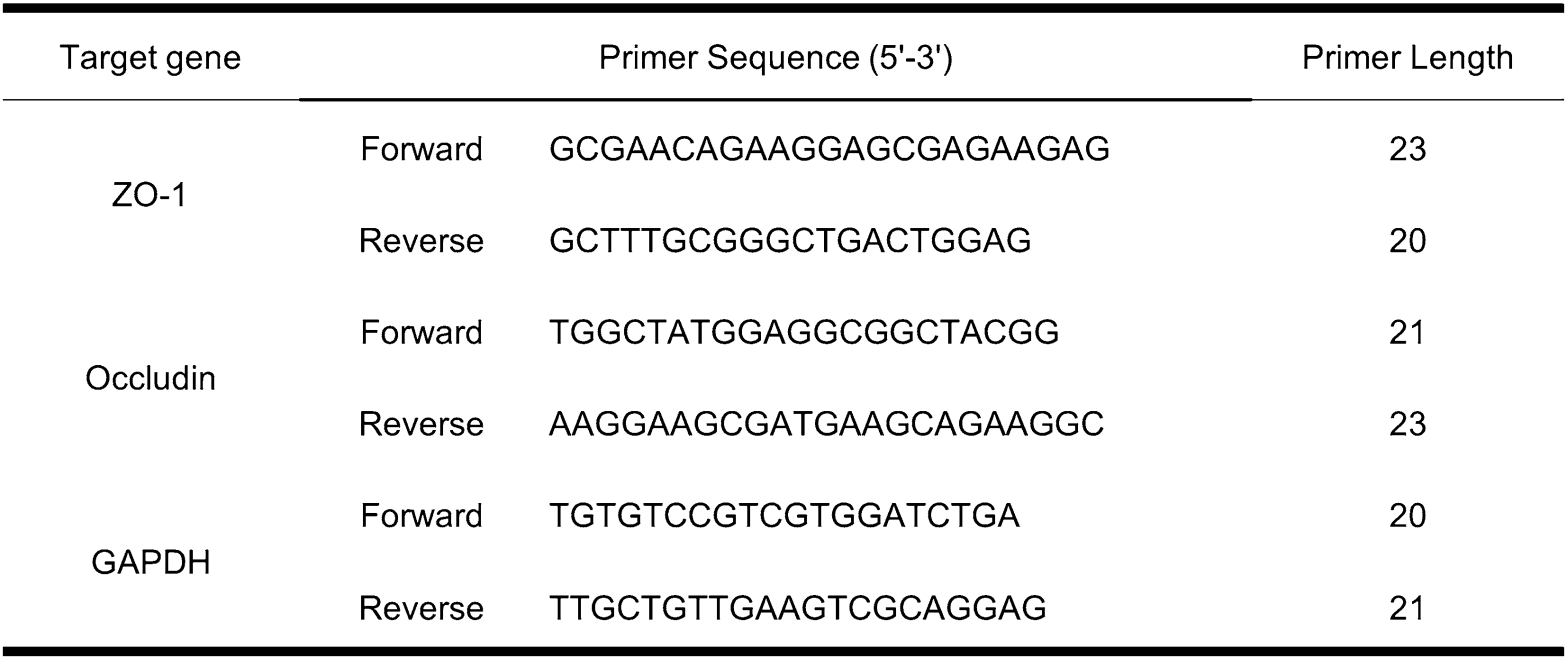
Primer sequences was used in RT-qPCR assays in colonic tissue.

### Measurement of cytokines in colon

The contents of tumor necrosis factor-alpha (TNF-α), interleukin-6 (IL-6) and interleukin-1 beta (IL-1β) in full colons were measured using the bicinchoninic acid (BCA, P0010S) protein assay kit (Beyotime Biotechnology, China) and Enzyme-Linked Immunosorbent Assay (ELISA) kits (Dakowei Biotechnology, Beijing, China) according to our previous method^61^.

### Western blot

The protein in colon was added to the corresponding proportion of SDS gel loading buffer and boiled for 5 min. After SDS-PAGE electrophoresis and transferred membrane, the membrane was blocked 5% skim milk in TBST buffer, at room temperature for 1 h and washed with TBST 3 times. Then β-actin (1:10,000, AC026, ABclonal, China), NF-κB (1:2,000, A11204, ABclonal, China) primary antibodies were added, and the membranes were incubated overnight at 4 °C, washed 3 times with TBST. Secondary antibodies conjugated with HRP against either rabbit or mouse IgG (1:2, 000, AS014, and AS029, ABclonal, China) were incubated for 1 h at 4 °C. The membrane was washed 3 times and enhanced chemiluminescence (ECL) developed. The gel imaging system (Amersham Imager 600, America) was photographed.

### DNA extraction and 16S rRNA gene amplification (Illumina MiSeq Sequencing)

Total bacterial genomic DNA was extracted from the cecum contents of mice. DNA was quantified and detected using NanoDrop2000 and 1% agarose gel electrophoresis, respectively. The V3-V4 region of the 16S rRNA gene was amplified by PCR using TransStart^®^ FastPfu DNA Polymerase. PCR amplification was performed with primers (338F: 5ʹ-ACTCCTACGGGAGGCAGCAG-3ʹ and 806R: 5ʹ-GGACTACHVGGGTWTCTAAT-3ʹ). PCR amplification products were separated by 2% agarose gel electrophoresis. The DNA library was constructed using the TruSeq Nano DNA LT Library Prep Kit by the Illumina MiSeq. The optimized library was verified using the Agilent High Sensitivity DNA Kit and then used for sequencing by the Illumina MiSeq. The Illumina MiSeq platform was used for community DNA fragment Paired-end sequencing. The sequencing results came from Beijing Microeco Technology Co., Ltd.

### Determination of contents of SCFAs

The contents of SCFAs including acetic, propionic and butyric in colon contents were determined by gas chromatography/mass spectrometry (GCMS) (Agilent 7010B, America) with calculating internal standard method. Before GCMS analysis, the appropriate amount of colon contents was mixed with 5 times the volume of water by vortexing and centrifuged at 11800 rpm for 10 min at 4 °C. The clear supernatant 200 μl was mixed and intermittent vortexed for 3 min with 20 μl volume of 2-ethylbutyric acid (internal standard), 500 μl concentrated hydrochloric acid (HCl) and 2 ml diethyl ether. The clear supernatant was transferred and added anhydrous sodium sulfate (Na_2_SO_4_), and intermittent vortexed for 2 min. The supernatant was taken and filtered with the 0.22 μm filter membrane after standing. Agilent J&W DB-FFAP (30 m × 0.25 mm × 0.25 μm) was used for SCFA separation. The temperatures of the inlet, ion source and transfer line were all set to 240 °C. The column temperature was programmed with an initial temperature of 100 °C for 0.5 min, 8 °C/min to 200 °C hold 2 min, and 10 °C/min to 240 °C hold 1 min. The energy of electron ionization (EI) was set to 70 eV.

### Statistical analysis

All data are expressed as the mean ± standard deviations (SD). Significant differences between two groups were assessed by using the Student's t test. Comparison between multiple groups was analyzed by using the one-way ANOVA test. *p* < 0.05 was considered to indicate a statistically significant difference.

### Ethics approval and consent to participate

All animal experiments complied with the ARRIVE guidelines and were carried out following the U.K. Animals (Scientific Procedures) Act, 1986 and associated guidelines, EU Directive 2010/63/EU for animal experiments, the National Institutes of Health guide for the care and use of Laboratory Animals (NIH Publications No. 8023, revised 1978) and Use of Laboratory Animals and the Chinese Legislation on Laboratory Animals. The animal experimental protocol complied with the Animal Management Rules of the Chinese Ministry of Health (document no. 55, 2001) and was approved by the Animal Experiment Ethics Committee of Qilu University of Technology.

## Supplementary Information

Supplementary Figures.
