# Supplementary material for: Lentinan improves intestinal inflammation and gut dysbiosis in antibiotics-induced mice
Source: Sci Rep. 2022 Nov 15;12:19609. doi: 10.1038/s41598-022-23469-2 (PMC9666428; doi:10.1038/s41598-022-23469-2)

**Supplementary Figure Legends**

**Supplementary Figure 1.** The colon length and ileum length of mice. Different groups by one-way ANOVA procedure followed by Duncan test. All error bars are SD. Nonsignificant difference is not marked on the graph.

**Supplementary Figure 2.** Comparison of alpha diversity for mice. Shannon index and Observed otus index. Different groups by one-way ANOVA procedure followed by Duncan test. All error bars are SD. Values with different letters are significantly different (*p* < 0.05). If one group and the other group are respectively marked different letters as a, b or c, it means significant differences between these two groups.

**Supplementary Figure 3.** Comparison of Beta diversity for mice. Unweighted UniFrac Principal coordinate analysis (Unweighted UniFrac PCoA) and Weighted UniFrac Principal coordinate analysis (Weighted UniFrac PCoA).

**Supplementary Figure 4.** Relative abundance of the Phyla level. Different groups by one-way ANOVA procedure followed by Duncan test. Values with different letters are significantly different (*p* < 0.05). All error bars are SD. If one group and the other group are respectively marked different letters as a, b or c, it means significant differences between these two groups.

**Supplementary Figure 5.** Original, unprocessed and full-length blots/gels.

**Supplementary Figure 1:**

**
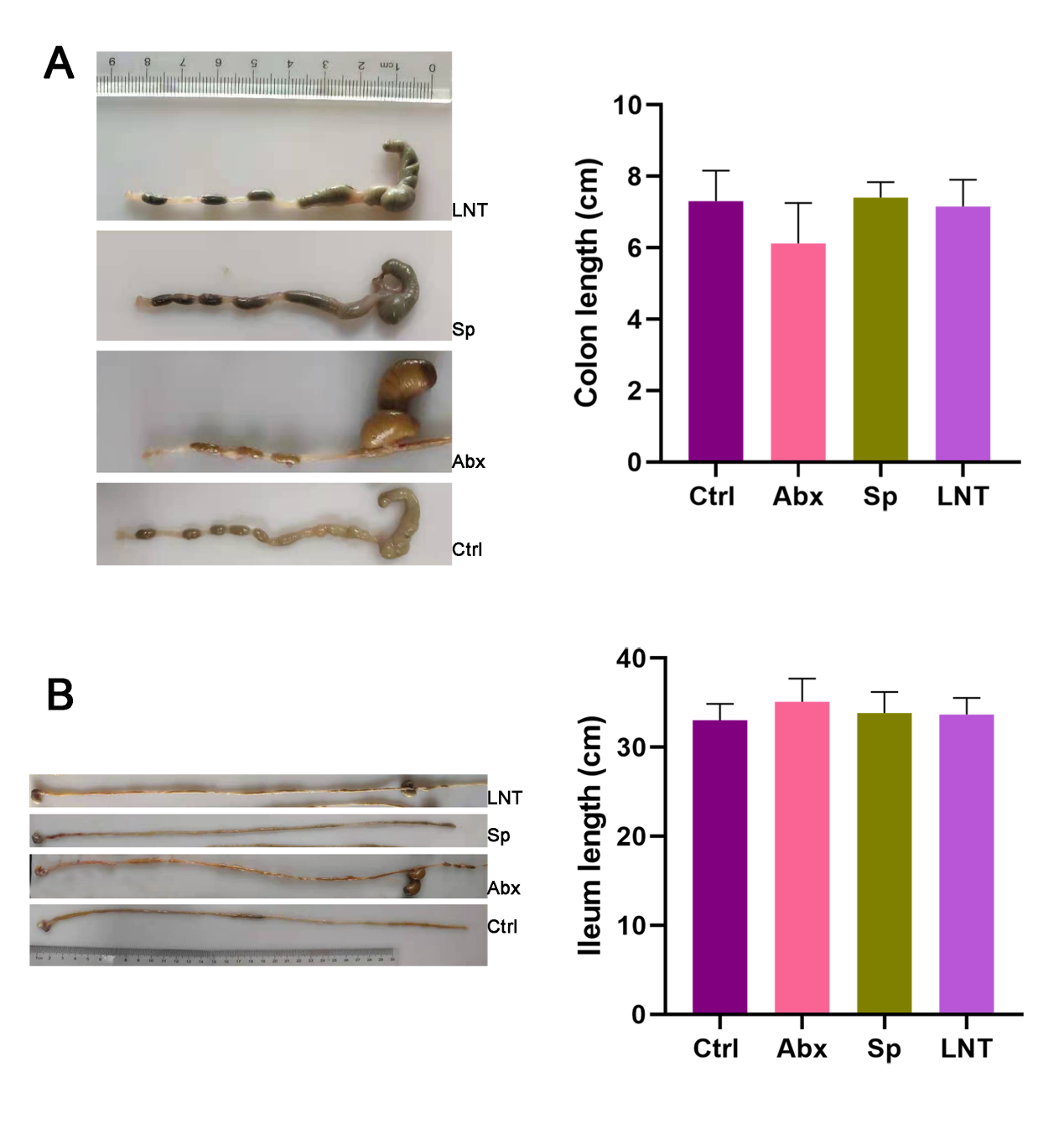
**

**Supplementary Figure 2:**

**
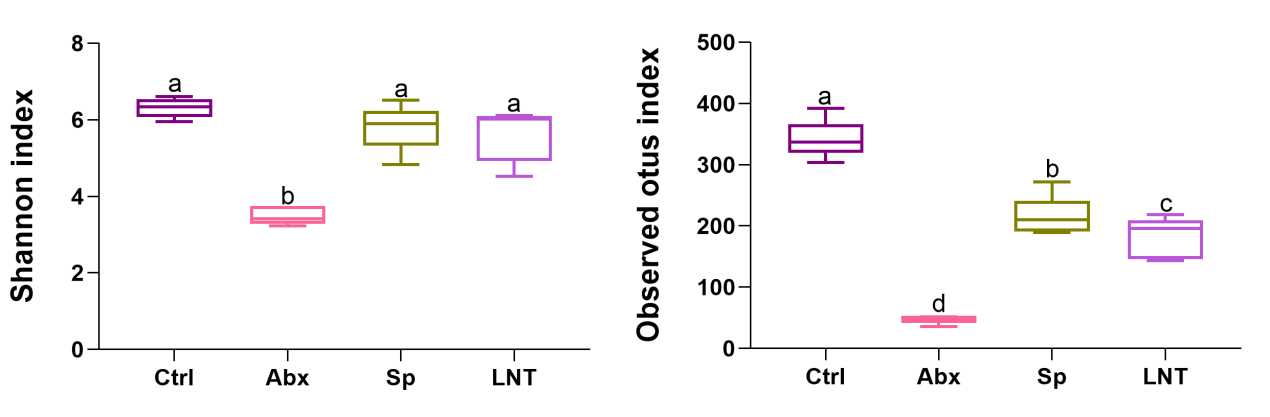
**

**Supplementary Figure 3:**

**
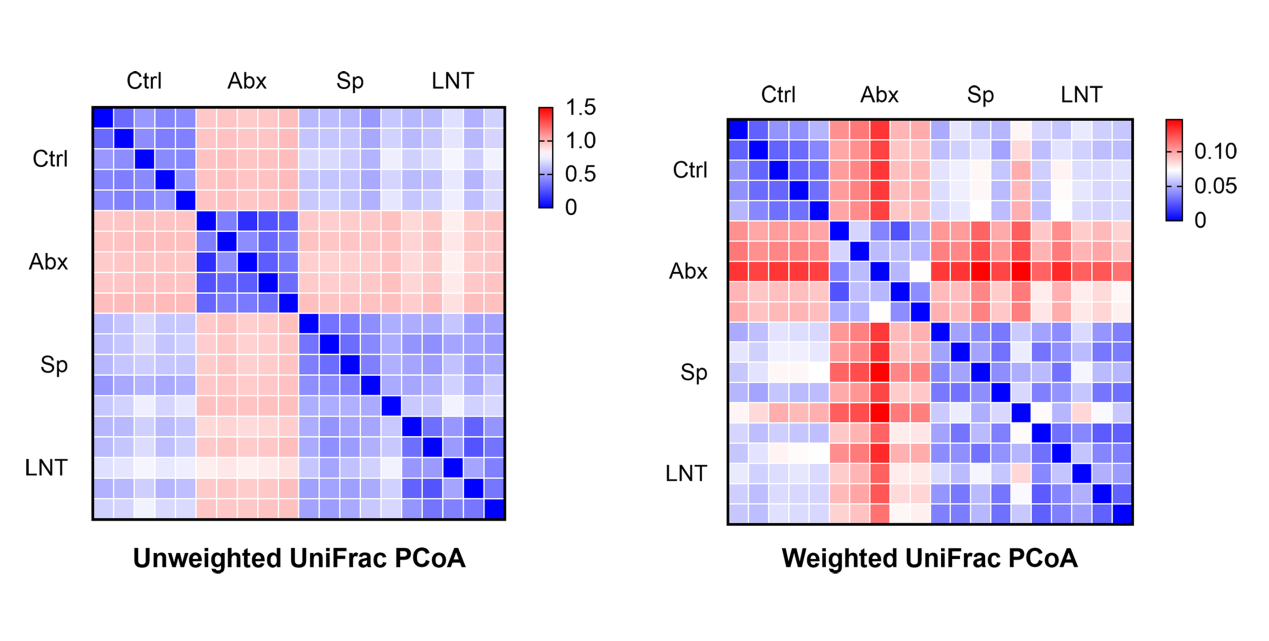
**

**Supplementary Figure 4:**

**
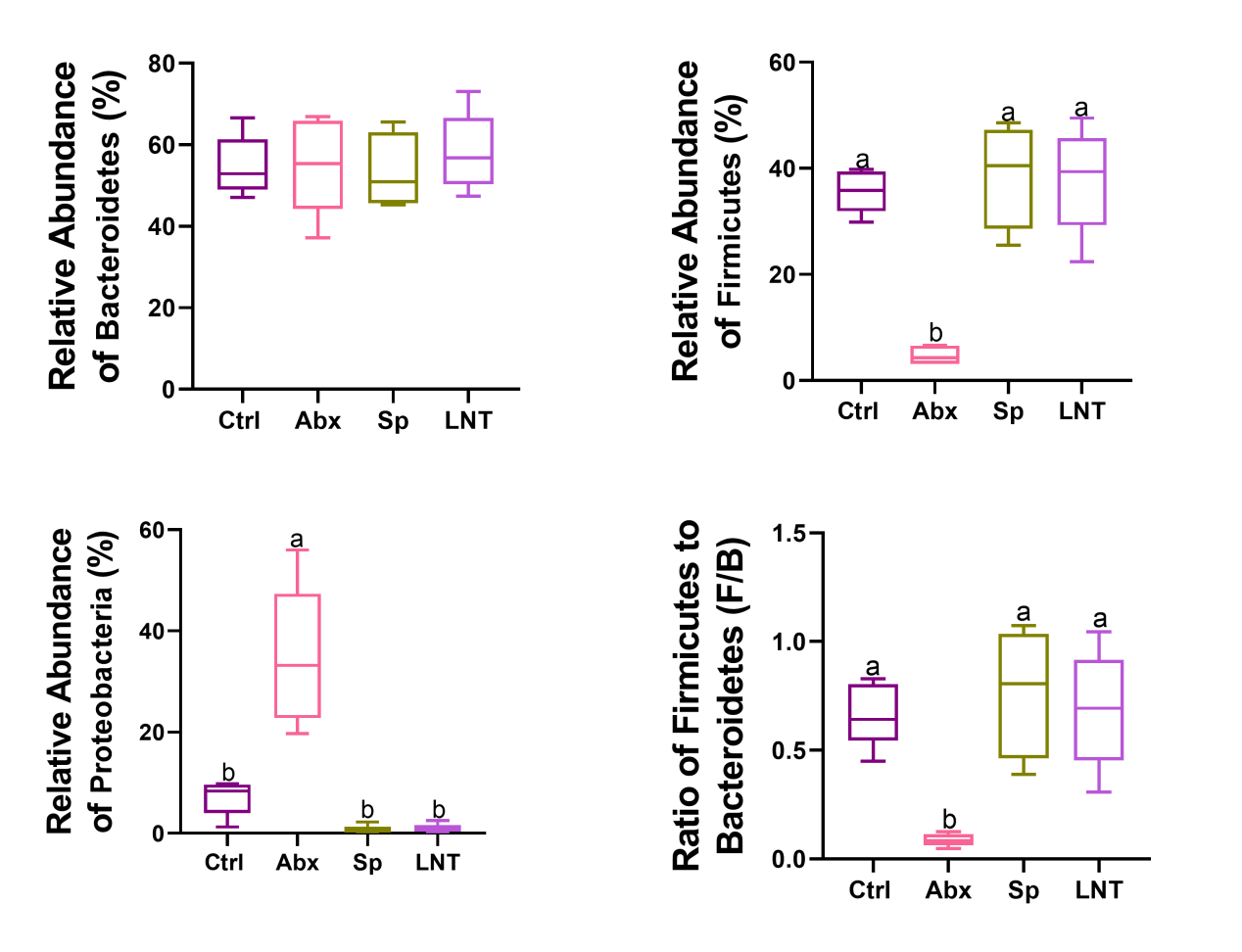
**

**Supplementary Figure 5:**


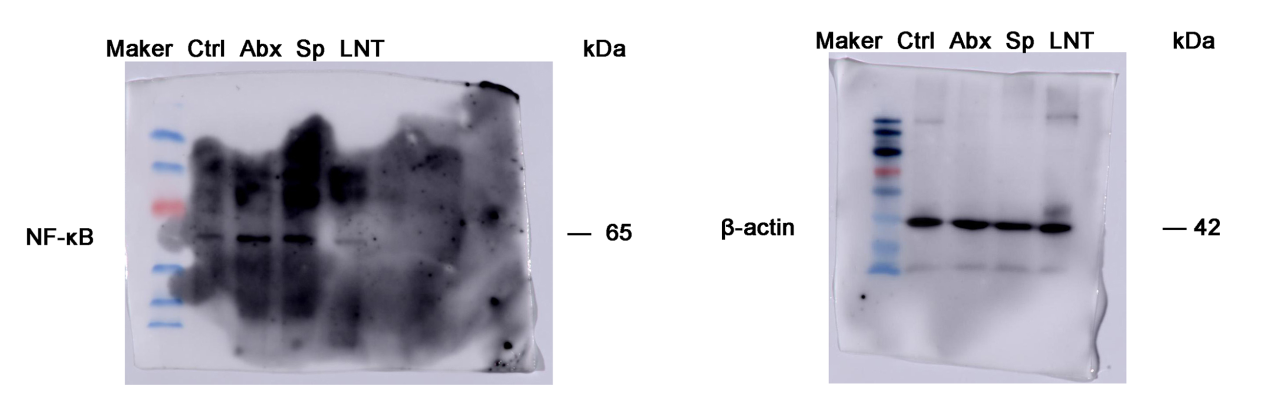

Supplement: Supplementary file 1 — Supplementary Figures. [file 41598_2022_23469_MOESM1_ESM.docx]
